# Colocalisation of CD9 and mortalin in CD9-induced mitotic catastrophe in human prostate cancer cells

**DOI:** 10.1038/sj.bjc.6603964

**Published:** 2007-09-11

**Authors:** V Zvereff, J-C Wang, K Shun, J Lacoste, M Chevrette

**Affiliations:** 1 Division of Experimental Medicine, Department of Medicine, McGill University, Montreal, Quebec, Canada; 2 Lady Davis Institute, Jewish General Hospital, McGill University, Montréal Quebec, Canada; 3 Division of Urology, Department of Surgery, McGill University and the Research Institute of the McGill University Health Centre, Montreal, Quebec, Canada

**Keywords:** CD9, mortalin, prostate, senescence, mitotic catastrophe

## Abstract

CD9, a member of the tetraspanin family of proteins, is involved in a variety of cellular interactions with many other proteins and molecules. Although CD9 has been implicated in cell fusion, migration and cancer progression, the detailed function of this protein is not completely understood and likely depends on interactions with different protein partners, which are not yet all known. Using co-immunoprecipitation and mass-spectrometric protein sequencing, we have identified in prostate cancer cells, a novel CD9 partner, the 75-kDa protein HSPA9B, also known as mortalin. We further show that introduction and overexpression of wild-type CD9 into human PC-3 prostate cancer cells induces mitotic catastrophe. We also demonstrate, by immunocolocalisation studies, the interaction of CD9 and mortalin in PC-3 cells undergoing mitotic catastrophe. Our results not only identified mortalin as a new CD9 partner, but also clarify the mechanisms by which CD9 may control prostate cancer progression.

The cluster-of-differentiation antigen 9 (CD9) protein belongs to the family of tetraspanin (also called transmembrane 4 superfamily or TM4SF) whose members have been implicated in cell motility and cell–cell interactions. In humans, the tetraspanin family is composed of at least 32 members (Boucheix *et al*, 2004). Many of these proteins (including CD9) are broadly expressed and form complexes with different partners, including other tetraspanins (Maecker *et al*, 1997), integrins (Berditchevski and Odintsova, 1999) and lineage-specific proteins (Hemler, 2001). Through these interactions and the formation of homodimers (Kovalenko *et al*, 2004), tetraspanins serve as building blocks in multicomponent protein complexes (Boucheix *et al*, 2004) and are considered to be ‘molecular facilitators’ (Maecker *et al*, 1997).

Cluster-of-differentiation antigen 9 (also called MRP1) was identified as the antigen binding to the mAb M31-15, an antigen found to inhibit the motility of various cancer cell lines (Miyake *et al*, 1991). Elevated CD9 expression is considered a favourable prognosticator in head and neck squamous cell carcinoma (Erovic *et al*, 2003). Cluster-of-differentiation antigen 9 is also expressed at higher levels in primary tumours as compared to metastatic human colon carcinoma cells (Cajot *et al*, 1997). Decreased CD9 expression has been associated with poor prognosis in breast (Miyake *et al*, 1996), non-small-cell lung cancer (Higashiyama *et al*, 1998), non-Hodgkin's lymphoma (Yau *et al*, 1998), and in oesophageal squamous cell carcinoma (Uchida *et al*, 1999). Exogenous expression of CD9 suppressed motility of mouse melanoma cells, and reduced their metastatic potential (Ikeyama *et al*, 1993). Our recent studies have shown that CD9 expression is also significantly reduced and even lost during prostate cancer progression (Wang *et al*, 2007). In contrast, in gastric cancers, CD9 facilitates cell proliferation through enhancement of the mitogenic activity of HB–EGF and consequently influences cancerous cell proliferation or metastasis generation (Hori *et al*, 2004). Moreover, exogenous CD9 expression does not significantly affect the growth and the invasive properties of a highly metastatic prostate cancer cell line (Zvieriev *et al*, 2005).

The current work was undertaken to understand the phenotypic differences observed following expression of CD9 in prostate tumour cell lines having increased metastatic potential. We show that overexpression of CD9 in human PC-3 prostate cancer cells is sufficient to induce mitotic catastrophe, which is also known as mitotic cell death. In contrast, overexpression of CD9 in PC-3 variants, obtained from serial *in vivo* selection using orthotopic implantation in nude mice and having increased metastatic potential (Pettaway *et al*, 1996), did not induce mitotic catastrophe (Zvieriev *et al*, 2005). Since CD9 is known to interact with other tetraspanins as well as other proteins involved in different cellular functions, the differences in phenotypic changes brought by CD9 expression in various types and aggressiveness of cancer cells are likely to depend on the presence or absence of different CD9 partners.

In order to characterise such partners in prostate cancer cells, we used mass-spectrometric analysis to identify proteins that co-immunoprecipitated with CD9. One of these proteins was the HSPA9B protein, a member of the heat-shock protein 70 (hsp70) family also known as mortalin. Mortalin, a putative oncogene product, has been implicated in many biological processes such as control of cell proliferation (Kaul *et al*, 2000b), differentiation (Xu *et al*, 1999) and tumorigenesis (Takano *et al*, 1997). Although mortalin is ubiquitously expressed, differential cellular localisation of mortalin in human cancer cells has been associated with cell senescence (Wadhwa *et al*, 1995). Indeed while in normal cells, mortalin is evenly distributed throughout the whole cytoplasm, in cancer cells it varies from fibrous or granular perinuclear localisation to a pan-cytoplasmic gradient. Here, we show that mortalin is not only associated with CD9, but also immunolocalised with CD9 when cells undergo CD9-induced mitotic catastrophe.

## MATERIALS AND METHODS

### Cell culture and transfections

The human PC-3 prostate cancer cell line, originally established from a bone metastasis of a grade IV prostate cancer (Kaighn *et al*, 1979), was obtained from the American Type Culture Collection (Manassas, VA, USA). The PC-3M cell line was derived from a liver metastasis of PC-3 cells following intraspleenic injection of these cells in nude mice (Pettaway *et al*, 1996). Sequential orthotopic injections of PC-3M cells into the prostate of nude mice generated a very aggressive prostate-growing cell line (the prostate-selective PC-3M-Pro4) and a highly migrating lymph node metastatic cell line (the lymph node-selective PC-3M-LN4) (Pettaway *et al*, 1996). PC-3M, PC-3M-Pro4 and PC-3M-LN4 were kindly provided by IJ Fidler (The University of Texas MD Anderson Cancer Center, Houston, TX, USA). The DU 145 cell line, originally derived from a prostate cancer brain metastasis (Stone *et al*, 1978), was obtained from the American Type Culture Collection. Cells were cultured in RPMI-1640 medium (Canadian Life Technologies, Burlington, ON, Canada) supplemented with 10% fetal bovine serum (Canadian Life Technologies). All cell lines were grown in the absence of antibiotics in a humidified incubator at 37°C and 5% CO_2_ and were free of mycoplasma as determined by Hoechst 33342 staining (Chen, 1977).

Generation of CD9 expression vector was described elsewhere (Zvieriev *et al*, 2005). PC-3, PC-3M, PC-3M-Pro4, PC-3M-LN4 and DU 145 cells were stably transfected with pcDNA3.1/Myc-HisA-CD9 wild-type construct using SuperFect™ transfection reagent (Qiagen, Mississauga, ON, Canada) following the manufacturer's instructions. Stable clones were selected in media containing 1000 *μ*g ml^−1^ of G418 (Canadian Life Technologies) and further maintained in the presence of 400 *μ*g ml^−1^ of G418. PC-3CD9 overexpressing clones were also obtained from a pool population of CD9-transfected PC-3 cells. Diluted single-cell suspensions of CD9-transfected PC-3 cells were seeded in Petri dishes (30–50 cells per dish). After 21 days in culture, individual CD9-transfected clones containing 200–500 cells were isolated.

### Immunoprecipitation and immunoblotting

Anti-CD9 (MM2/57) and anti-actin (CBL171) mAbs were obtained from Chemicon International (Temecula, CA, USA), anti-mortalin mAb (MA3-028) was purchased from eBiosciences (San-Diego, CA, USA), and HRP-coupled goat anti-mouse secondary antibody (no. 31430) was from Pierce Biotechnology (Rockford, IL, USA). Non-immune mouse IgG was purchased from Sigma-Aldrich Canada Ltd (Oakville, ON, Canada).

For immunoprecipitation and immunoblotting studies, cells were grown in 100 mm Petri dishes and harvested in lysis buffer containing 1% Brij^©^ 97, 25 mM HEPES, pH 7.5, 150 mM NaCl, 5 mM MgCl_2_, 2 mM PMSF, 10 *μ*g ml^−1^ aprotinin and 10 *μ*g ml^−1^ leupeptin. For co-immunoprecipitation experiments, protein extracts (1 mg) were precleared and incubated overnight with a specific CD9 mAb (2 *μ*g of MM2/57) and protein Sepharose G beads (1 : 1 slurry, GE Healthcare, Baie d’Urfe, QC, Canada). Immunocomplexes were collected by boiling in Laemmli buffer, loaded onto SDS-PAGE and run under non-reducing conditions. Silver staining was used to reveal proteins obtained from CD9 immunocomplexes. Bands were excised, digested with proteases and sequenced using matrix-assisted laser desorption/ionisation time-of-flight mass spectrometry (MALDI-TOF MS at the proteomics platform of McGill University and Genome Quebec Innovation Centre, Montreal, QC, Canada). For immunoblotting, following SDS-PAGE (run under reducing condition for mortalin detection and non-reducing for CD9 detection), the proteins (50 *μ*g) were transferred to Trans-Blot Transfer Medium Membrane (Bio-Rad Laboratories, Hercules, CA, USA) and blocked in 5% non-fat dry milk. Protein bands were detected with mortalin primary mAb (1 : 500 dilution), or CD9 primary mAb (1 : 500 dilution). Signal was amplified with HRP-coupled goat anti-mouse secondary antibody (1 : 2000 dilution) and detected with a chemiluminescent kit (Amersham, Oakville, ON, Canada). Actin detection (MAB1501R; 1 : 2000 dilution) was performed to confirm equal protein sample loading.

### Immunofluorescence and colocalisation studies

Cells seeded onto glass coverslips in six wells were fixed in 3.7% paraformaldehyde following incubation in 50 mM of ammonium chloride, and permeabilised in 0.2% (v v^−1^) Triton X-100. Signal detection was obtained by a mixture of primary antibodies (1 : 50 dilution for mortalin antibody; 1 : 300 for CD9 antibody). In co-immunostaining experiments, goat anti-mouse conjugated Alexa 555 and goat anti-mouse conjugated Alexa 488 (Invitrogen, Burlington, ON, Canada) were used as fluorochromes. The coverslips were inverted on a glass slide and treated with Immu-Mount mounting medium (ThermoShandon, Pittsburgh, PA, USA).

Cells were imaged using a Zeiss LSM 5 Pascal confocal microscope (Carl Zeiss International, Toronto, ON, Canada) with a × 100/1.3 objective and an argon laser (488 nm). Images were processed using the Axiovision (Carl Zeiss International) and Adobe Photoshop (Adobe Systems, Mountain View, CA, USA) programs.

### Immunocytochemistry

A total of 2–4 × 10^4^ cells were seeded onto coverslips in a 12-well plate and incubated overnight at 37°C. The next day, adherent cells were fixed with 3% of prechilled formaldehyde and ice-cold methanol. A section of benign prostatic hyperplasia (BPH) from transurethral resections of the prostate served as an external positive control for each immunocytochemistry staining. Before staining, endogenous peroxidase was removed by 3% solution of hydrogen peroxide (H_2_O_2_; Fisher Scientific, Nepean, ON, Canada) in 50% methanol (Fisher Scientific). The Histostain™-SP kit (Zymed, South San Francisco, CA, USA) was used to detect and amplify the signal originating from the binding of antibody to CD9 antigen. Cluster-of-differentiation antigen 9 protein expression was detected with monoclonal mouse anti-human CD9 antibody (NCL-CD9; 1/300 dilution; Novocastra Laboratories, Burlingame, CA, USA). The staining signal was revealed upon incubation with DAB substrate, prepared by dissolving Fast DAB tablets (Sigma-Aldrich Canada Ltd) in ddH_2_O. Cells were counterstained with haematoxylin and dehydrated in increasing graded ethanol and toluene. The coverslips were inverted on glass slides and sealed with Cytoseal 60 mounting solution (Richard-Allan, Kalamazoo, MI, USA).

## RESULTS

### Wild-type CD9 overexpression induces mitotic catastrophe in PC-3 prostate cancer cells

**Figure 1:**
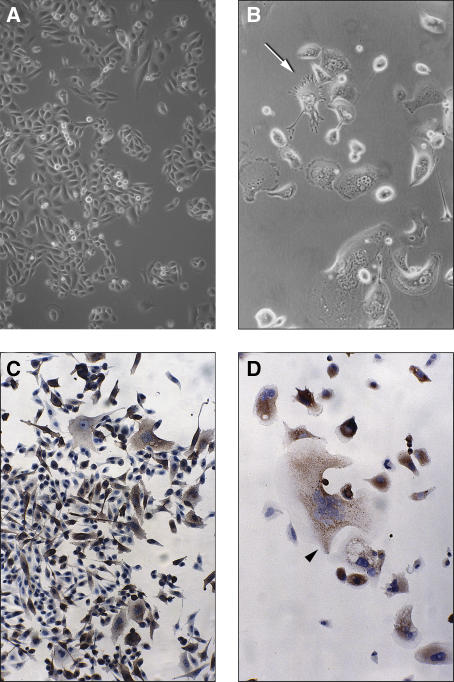
Cluster-of-differentiation antigen 9 (CD9) overexpression induces mitotic catastrophe in PC-3 cells. Morphology of PC-3 (untransfected) and CD9-transfected PC-3 cells, photographed under phase-contrast microscope. (**A**) PC-3 (untransfected) cell morphology showing the presence of rounded, dividing cells at passage 20. (**B**) Cells with significant morphological changes are shown here for the PC-3CD9-69 clone at passage 1. An arrow indicates a cell with characteristic spindle-like cellular projections. (**C**) A mixture of prominent enlarged cells and small spindle-shaped cells of PC-3MCD9-16 clone at passage 1. Immunocytochemical staining revealed that all enlarged cells highly express CD9 protein, whereas most small, spindle-shaped cells expressed a very low amount (if any) of CD9. These CD9-negative cells will gradually become the dominant growing population. (**D**) Picture of an exaggerated enlarged cell with CD9 staining from PC-3CD9-18 at passage 0 (arrowhead). Break down of cytoskeleton is visible in the cell just below it. Most cells have an intense and diffuse CD9-staining pattern, occasionally with punctuate distribution. (Original magnification: **A**–**D**, × 100)

We had already demonstrated that as compared to BPH, the CD9 protein is expressed at lower level in human prostate cancer cell lines (data not shown). Therefore, to characterise further the role played by CD9 in prostate cancer, we stably transfected and overexpressed wild-type CD9 in PC-3 cells and its derivatives (all originally obtained upon injection in nude mice), namely PC-3M (a liver metastasis obtained upon PC-3 intraspleenic injection), PC-3M-Pro4 (a highly aggressive cancer cell line selected upon sequential orthotopic PC-3M injections) and PC-3M-LN4 (a very aggressive lymph node metastatic cell line selected upon sequential orthotopic PC-3M injection). While cultured PC-3 cells appear as small and rounded (Figure 1A), in contrast, CD9-transfected PC-3 cells underwent drastic morphological changes (Figure 1B and D). Characteristic appearances of CD9-transfected cells included an enlarged cell surface, prominent cellular projections (indicated by an arrow in Figure 1B), and multiple nuclei (Figure 1B and as shown by an arrowhead in Figure 1D). Compared to parental PC-3 cells (Figure 1A), the cell surface of some CD9-transfected cells increased by as much as 10-fold (Figure 1B and D). Many intracytoplasmic vacuoles were seen in those enlarged cells (Figure 1B). Cluster-of-differentiation antigen 9-overexpressing PC-3 cells also had a limited lifetime and usually reached less than 10% of cell confluence. Such morphological changes were also seen in some CD9-transfected PC-3M cells (Figure 1C). As these phenotypic changes became more severe, fewer cells appeared to be in mitosis. Eventually, all cells exhibited pronounced phenotypic changes and overall less than 10 cells remained in the flask. Such characteristics are hallmarks of a particular type of cell death called mitotic catastrophe, which Roninson *et al* (2001) defined as a type of cell death resulting from abnormal mitosis ending in the formation of large cells with multiple micronuclei and decondensed chromatin. These morphological changes also contrast with the shrunken cytoplasm and the condensed nuclei seen when cells undergo apoptosis (Roninson *et al*, 2001). We have detected a low percentage of such apoptotic cells in some CD9-transfected cells that either underwent or not mitotic catastrophe. Although mitotic catastrophe is sometimes followed by apoptosis (Jordan *et al*, 1996), it is not required for the lethal effect of mitotic catastrophe. It thus seems that CD9-induced mitotic catastrophe is not followed by apoptosis, a result consistent with the p53-null status of PC-3 cells (Isaacs *et al*, 1991), and with the reported apoptosis-reluctant status of PC-3M (a derivative of PC-3) cells (Demidenko and Blagosklonny, 2004).

We followed the fate of 61 CD-9-transfected PC-3 clones and found that 33% underwent mitotic catastrophe (Table 1). In contrast, CD9-transfected cells from the remaining two-thirds of PC-3 clones, 91% of PC-3M clones and 100% of PC-3M-Pro4, PC-3M-LN4 and DU 145 clones did not show any sign of mitotic catastrophe (Table 1) and could be kept more than 7 months in culture. We also obtained 57 ‘mock-transfected’ clones (containing pcDNA3.1A vector). Among these clones, 25 were derived from PC-3 cells, and none exhibited mitotic catastrophe (Table 1).

**Figure 2:**
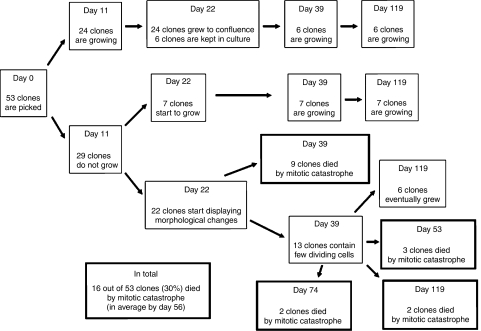
Growth characteristics and induction of mitotic catastrophe in cluster-of-differentiation antigen 9 (CD9)-transfected PC-3 clones. Fifty-three CD9-transfected PC-3 clones were monitored weekly. Six of the 24 clones showing strong growth capabilities were cultured for 119 days. Sixteen clones that had a limited lifetime were followed until mitotic catastrophe. In summary, 16 of 53 clones (30%) experienced mitotic catastrophe on average after 56 days of cell culture.

Although individual CD9-transfected PC-3 clones were isolated and expanded, many more were obtained and kept as pools. Since CD9-transfected clones eventually died, we subcloned the pooled population (at passage 2) to generate more CD9-transfected PC-3 clones. Fifty-three clones were picked and seeded into two T25 flasks (day 0). This allowed us to better define the time course of the induction of mitotic catastrophe in these cells (described in Figure 2). Clones were observed under the microscope and results were recorded on a weekly basis. Growth differences were observed by day 11. Out of the 53 clones picked, only 24 clones grew well and were cultured for many passages. Six clones from this series were maintained for more than 3 months. On the other hand, 7 of the 29 clones that did not originally grow started to grow by day 22. At the same time, moderate morphological changes, such as presence of enlarged cells, became evident in 22 of the 29 non-growing clones; these cells eventually experienced mitotic catastrophe. By day 39, nine clones were no longer viable. In the 13 remaining clones, isolated colonies started to grow by days 43–46 and viable cultures were obtained from six of them. The remaining seven clones eventually died at day 53 (three clones), day 74 and day 119 (two clones each). Overall, CD9 was able to induce mitotic catastrophe in 16 of 53 clones (30%) after approximately 56 days of culture. The percentage of CD9-transfected cells undergoing mitotic catastrophe in this subset of clones was very similar to the one obtained in the original selection, where 20 of 61 PC-3 CD9 clones (33%) died after 48 days in culture (Table 1). Indeed, there is no statistical difference between both sets of CD9-transfected clones undergoing mitotic catastrophe (*P*=0.84 by Fisher's exact test). However, such difference (30 and 33 *vs* 0%) in the number of clones undergoing mitotic catastrophe between CD9- and vector-transfected PC-3 cells is highly significant (*P*=0.0017).

**Figure 3:**
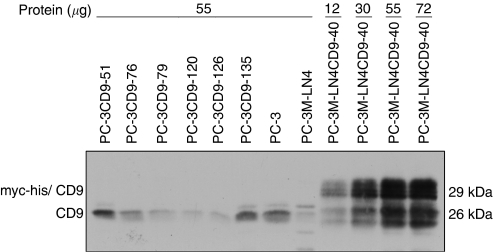
Lack of exogenous cluster-of-differentiation antigen 9 (CD9) expression in surviving PC-3-transfected cells. Detection of CD9 in prostate cancer cells by Western immunoblot using CD9 monoclonal antibody. Protein extracts (55 *μ*g) from six surviving CD9-transfected PC-3 clones, from PC-3 and from PC-3M-LN4 cells are compared to different amount of protein extracts (12–72 *μ*g) from PC-3M-LN4CD9-40 cells expressing exogenous CD9.

Cluster-of-differentiation antigen 9 immunocytological staining was performed on 50% (57 of 114) CD9-transfected PC-3 clones. Since most dying clones could not be divided to allow immunocytochemistry staining, we were only able to stain 5 out of 36 clones (from both sets of clones). Immunocytochemical analysis showed that all five dying clones were CD9-positive. In contrast, only 14 (27%) of the 52 growing clones were CD9-positive. Thus, the majority (73%) of these clones had lost exogenous CD9 expression. Moreover, most growing CD9-positive clones experienced a gradual reduction of CD9 expression, as seen by lower CD9-staining intensity and decreasing population of CD9-positive cells in culture. The difference in CD9 expression between dying and growing clones was highly significant using either *χ*^2^ test (*P*=0.0009) or Fisher's exact test (*P*=0.003). This lack of exogenous CD9 expression in surviving CD9-transfected clones was confirmed by immunoblotting (Figure 3). These results show that when CD9-transfected PC-3 cells are kept in culture, exogenous CD9 protein expression was silenced, allowing the growth of these cells.

### Identification of CD9-associated protein

**Figure 4:**
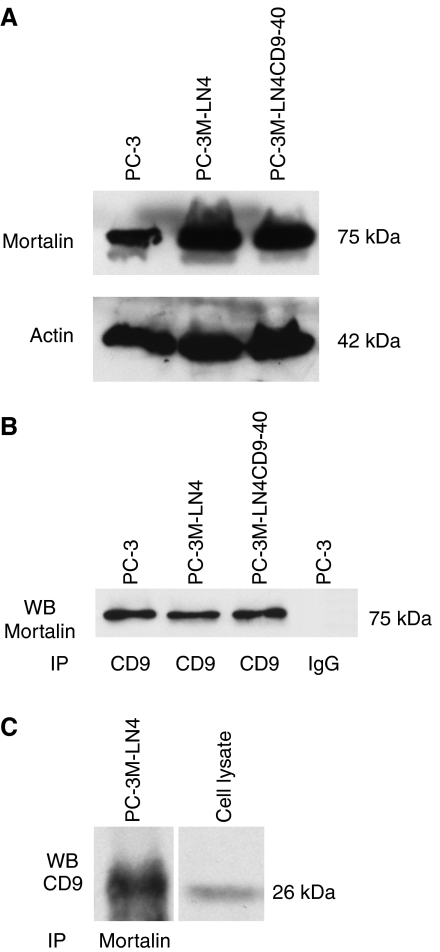
Analysis of mortalin expression in prostate cancer cells. (**A**) Detection of mortalin in prostate cancer cells by Western immunoblot using mortalin monoclonal antibody. Actin detection was performed to confirm equal protein sample loading. (**B**) PC-3, PC-3M-LN4 and PC-3M-LN4CD9-40 cells were lysed in 1% Brij 97, protein extracts were immunoprecipitated with either cluster-of-differentiation antigen 9 (CD9) mAb or IgG, separated on SDS-PAGE, transferred to nylon membrane and revealed with mortalin mAb. Immunoprecipitation with non-specific IgG (mouse) was performed for non-specific band detection. (**C**) PC-3 cells were lysed in 1% Brij 97, protein extracts were immunoprecipitated with mortalin mAb, separated on SDS-PAGE, transferred to nylon membrane and revealed with CD9 mAb. Cell lysate from PC-3M-LN4 cells was loaded as a control for CD9 detection.

Like other tetraspanins, CD9 interacts with many partners, such as integrins and other tetraspanins (Hemler, 2001; Charrin *et al*, 2003). Indeed, more than 30 CD9 partners have recently been identified in colon cancer cells; CD9 partners even differ between cell lines established from the primary tumour and the metastases obtained from a unique patient (Le Naour *et al*, 2006). We hypothesised that differential expression or distribution of such partners could be responsible for the striking differences observed following CD9 overexpression in the different human prostate cancer cell lines (Table 1). Immunoprecipitation was thus used in an attempt to identify such partners. For these studies, PC-3 and PC-3M-LN4 cells were used since they respond differently to CD9 overexpression (Table 1). Cluster-of-differentiation antigen 9 immunocomplexes obtained from Brij 97 lysates of PC-3 and PC-3M-LN4 cells were fractionated by SDS-PAGE. Silver staining of the gel revealed a 75-kDa protein, which is clearly present in PC-3 and PC-3M-LN4 cells (data not shown). This protein was isolated and sequenced using mass spectrometry. Peptide sequences (Table 2) identified this protein as HSPA9B, a member of the hsp70 family, also known as mortalin, PBP74, mthsp70 or MOT. Using an anti-HSPA9B monoclonal antibody, we confirmed that mortalin is expressed in both PC-3 and PC-3M-LN4 cell lines (Figure 4A), regardless from the fact that CD9 overexpression had contrasting effects on their viability (Table 1). Furthermore, immunoprecipitation of cell lysates with an anti-CD9 antibody resulted in co-precipitation of mortalin (Figure 4B). Converse immunoprecipitation with anti-mortalin antibody revealed CD9 on immunoblot (Figure 4C), confirming their interaction in prostate cancer cells. Although not quantitative, we could not detect any significant difference in the amount of CD9 immunoprecipitated with the mortalin antibody in PC-3 and PC-3M-LN4 cells.

### Colocalisation of mortalin and CD9 in prostate cancer cells

**Figure 5:**
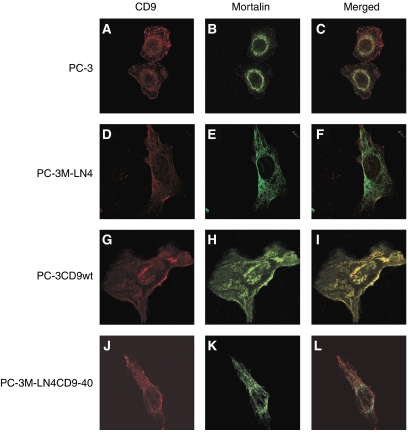
Immunofluorescence analysis of cluster-of-differentiation antigen 9 (CD9) and mortalin in prostate cancer cells. Confocal laser scanning micrographs show staining for CD9 (red signals: **A**, **D**, **G** and **J**) and mortalin antigens (green signals: **B**, **E**, **H** and **K**). Overlap of signals in merged images revealed colocalisation of CD9 and mortalin in cells undergoing mitotic catastrophe (yellow staining in panel **I**). No protein colocalisation is detected in the other images (**C**, **F** and **L**).

Earlier reports demonstrated that subcellular distribution of mortalin differed significantly in various cancer cells and allowed classification of cells into four senescence groups (Wadhwa *et al*, 1995). We compared the localisation of CD9 and mortalin by immunofluorescent staining in PC-3 and PC-3M-LN4 cells. In PC-3 cells, mortalin expression follows a perinuclear pattern (Figure 5B) whereas in PC-3M-LN4 cells, mortalin is seen as a gradient from the nucleus to the cell membrane (Figure 5E). In contrast, CD9 expression pattern was the same (a weak diffuse-typed cytoplasm with occasional cell membrane staining) in both cell lines (Figure 5A and D). Although these two proteins co-immunoprecipitated, confocal microscopy analysis failed to show colocalisation of the two proteins in these cells (Figure 5C and F). Since mortalin expression has been implicated in cell senescence and immortalisation (Kaul *et al*, 1998; Wadhwa *et al*, 2004), we determined if mortalin distribution was affected in CD9-transfected PC-3 cells undergoing mitotic catastrophe.

PC-3 cells were once again transfected with wild-type CD9 and 24 new PC-3CD9 clones were obtained. These cells were kept in culture and observed until (for the third time) signs of mitotic catastrophe became obvious. Double immunofluorescent staining with both anti-CD9 and anti-mortalin mAbs was then performed. Untransfected PC-3 and PC-3M-LN4 cells and PC-3M-LN4 cells overexpressing CD9 (PC-3M-LN4CD9-40 clone without mitotic catastrophe induction) were used as controls. Confocal microscopic analysis clearly showed that CD9 and mortalin colocalised in PC-3CD9 transfectants undergoing mitotic catastrophe (Figure 5I). Under these conditions, mortalin expression was switched from a perinuclear distribution (as in Figure 5B) to a pancytoplasmic localisation (Figure 5H), where it colocalised with CD9 (Figure 5I). These two proteins, although also expressed in the other cells analysed, did not colocalise in the absence of mitotic catastrophe (Figure 5C, F and L). Overexpression of CD9 in the more invasive PC-3M-LN4CD9-40 cells did not result in colocalisation of CD9 and mortalin (Figure 5L). Thus, CD9 and mortalin colocalisation seems to happen only when cells are undergoing mitotic catastrophe.

## DISCUSSION

Mitotic catastrophe is the main form of cell death induced by ionising radiation (Cohen-Jonathan *et al*, 1999) but can also be induced, albeit at low percentage, by different classes of cytotoxic agents such as etoposide (Lock and Stribinskiene, 1996), taxol (Torres and Horwitz, 1998), cisplatin or bleomycin (Tounekti *et al*, 1993). Since such treatments are currently used with more or less success against different types of cancer, there could be tremendous benefits in identifying genes that can induce mitotic catastrophe. By overexpressing CD9 in PC-3 cells, we demonstrated that this tetraspanin plays an active role in this process. Indeed, sustained overexpression of CD9 in these cells (maintained in a third of transfected clones) resulted in mitotic catastrophe, although at different times for different clones (Figure 2). Such delay is also a characteristic of mitotic catastrophe induction (Roninson *et al*, 2001), since cells first need to exit a growth arrest state and resume cell division for mitotic catastrophe to occur.

Using converse co-immunoprecipitation, we confirmed that mortalin is a new CD9 partner in prostate cancer cells. Mortalin is a highly conserved member of the hsp70 family of proteins, which was first identified by its presence in cytoplasmic fractions of normal mouse fibroblasts and its absence in similar fractions from immortal cells (Wadhwa *et al*, 1993a). Mortalin is predominantly present in mitochondria, but also reported in endoplasmic reticulum, cytoplasmic vesicles and cytosol (Ran *et al*, 2000). Mortalin is ubiquitously expressed, and so far has been detected in all mammalian cells analysed. This protein has been implicated in stress response (Ornatsky *et al*, 1995), muscle activity (Ibi *et al*, 1996), mitochondrial biogenesis (Schneider and Hood, 2000), control of cell proliferation (Kaul *et al*, 2000b), intracellular trafficking (Mizukoshi *et al*, 2001), differentiation (Xu *et al*, 1999) and tumorigenesis (Takahashi *et al*, 1994; Kaul *et al*, 1998; Wadhwa *et al*, 2006). Expression level of mortalin is frequently increased in tumours (Kaul *et al*, 2002; Dundas *et al*, 2005), where it is believed to cause inactivation of tumour suppressor protein TP53 (Wadhwa *et al*, 1998).

Immunofluorescence studies on mortalin localisation in more than 50 cell lines demonstrated differences in its distribution in normal and transformed human cells (Kaul *et al*, 2002). Indeed, in a study of cell senescence (Pereira-Smith and Smith, 1988), 30 immortal human cell lines were assigned to four complementation groups. Differences in pattern of mortalin distribution were later on associated with these four complementation groups (Wadhwa *et al*, 1995), highlighting mortalin involvement in senescence mechanisms. These observations, combined with the differential effects of CD9 overexpression in prostate cancer cells demonstrated in this study, led us to hypothesise that mortalin distribution in PC-3 and PC-3M-LN4 cells could also differ. Immunofluorescent studies demonstrated that the pattern of mortalin expression in PC-3 and PC-3M-LN4 cells was indeed different and thus classify these two closely related cell lines in two different senescence groups. PC-3 would thus belong to group C, with a granular–juxtanuclear arch mortalin staining, while PC-3M-LN4 cells, never showing such perinuclear staining, exhibited instead a granular gradient of mortalin expression from the nucleus to the cell membrane, defining PC-3M-LN4 as a member of group B (Wadhwa *et al*, 1995).

Although mortalin is present in CD9 immunoprecipitates from both PC-3 and PC-3M-LN4 cells, confocal analysis revealed that in cells, CD9 and mortalin colocalisation occurred only during CD9-induced mitotic catastrophe. These apparently contradictory results can be explained if one supposes that the perinuclear localisation of mortalin is essential to allow close contact between CD9 and mortalin. When cells are lysed, mortalin compartmentalisation is obviously released, allowing co-immunoprecipitation with CD9, confirming their interaction. Such importance for mortalin cellular localisation is also in agreement with the involvement of this protein in the different senescence groups (Wadhwa *et al*, 1995).

Our results also indicate that CD9 and mortalin interactions differ between PC-3 and PC-3M-LN4 cell lines. Although we cannot rule out that the strong interaction between CD9 and mortalin in PC-3 cells (seen in colocalisation studies, Figure 5I) could be due to a difference in the amount of proteins implicated in these complexes, taken together, our results argue that a perinuclear localisation of mortalin (as in PC-3 cells) is required for CD9-induced mitotic catastrophe and CD9/mortalin colocalisation. It is interesting to note that the importance of such differences in mortalin localisation was also seen with the two isoforms (mot-1 and mot-2) of mouse mortalin. Indeed, overexpression of the pancytoplasmic mot-1 protein is sufficient to induce cell senescence in NIH 3T3 mouse fibroblasts (Wadhwa *et al*, 1993b), while introduction of the mot-2 perinuclear mortalin induced their malignant transformation Kaul *et al* (1998). Since mouse mot-1 and mot-2 isoforms differ only by two amino acids (Kaul *et al*, 2000a), it is tempting to speculate that in human, where there is only one mortalin isoform, this dual mortalin function is insured by the binding of mortalin to specific partners, such as CD9. Perinuclar localisation of mortalin could thus not only define a senescence group (Wadhwa *et al*, 1995), but also identify cells or tumour types that will be sensitive to CD9 overexpression. If confirmed, such properties could eventually lead to a more targeted approach in cancer treatment.

The link between mortalin inactivation and cell senescence or cell death is very well established. Indeed, a reduction of mortalin expression using an RNA–helicase-coupled hybrid ribozyme resulted in cell growth arrest of HT1080 human fibrosarcoma cells (Wadhwa *et al*, 2003). Such reduction of mortalin was accompanied by an increase in TP53 expression (Wadhwa *et al*, 2003), which could indicate that mortalin interferes with the TP53 protein. Moreover, reduction of mortalin level by antisense RNA is sufficient to induce cell senescence, with the appearance of enlarged cells (Wadhwa *et al*, 2004). Reduction of mortalin expression with specific shRNA also resulted in growth arrest in human U2OS osteocarcinoma cells (Kaul *et al*, 2006). Our results will thus argue that the strong interaction and colocalisation of mortalin and CD9 seen in PC-3 cells results in inactivation of mortalin functions, which eventually lead to mitotic catastrophe. This phenotype, however, is not strictly dependent on wild-type TP53 functions, as PC-3 cells are not expressing this protein due to a frame-shift mutation (Isaacs *et al*, 1991).

In conclusion, we demonstrated that overexpression of CD9 in PC-3 cells creates appropriate conditions for CD9 and mortalin protein interactions, apparently by affecting distribution and/or expression of mortalin. Under such conditions, PC-3 cells undergo mitotic catastrophe. The ability to initiate cell death in cancer cells is very important to prevent unregulated cell growth. Our results showed that CD9 interactions with mortalin are linked to cell death through mitotic catastrophe in prostate cancer cells, showing the importance of CD9 in tumour suppression. Nevertheless, the mechanisms by which these two proteins can induce mitotic catastrophe are not clear and will require further elucidation.

## Figures and Tables

**Table 1:** Outcome of wild-type CD9 overexpression in human prostate cancer cell lines

**Prostate cancer cell lines^a^**	**No. of clones analysed**	**No. (percentage) of clones undergoing mitotic catastrophe**
DU 145	29	0 (0%)
PC-3	61	20 (33%)
PC-3^b^ (pcDNA3.1A)	25	0 (0%)
PC-3M	22	2 (9%)
PC-3M-Pro4	63	0 (0%)
PC-3M-LN4	39	0 (0%)

a DU 145 is a prostate patient-derived brain metastasis; PC-3 is a prostate patient-derived bone metastasis. PC-3 M, PC-3M-Pro4 and PC-3M-LN4 were obtained upon injection of cells in nude mice. PC-3M was derived from a liver metastasis of PC-3 intraspleenic injection; both PC-3M-Pro4 (growing very aggressively in the prostate) and PC-3M-LN4 (rapidly forming lymph node metastasis) were obtained upon sequential orthotopic injections of PC-3M cells.

b PC-3 (pcDNA3.1A) represents stable PC-3 clones analysed upon transfection with the empty vector (pcDNA3.1A).

**Table 2:** Sequence of peptides derived from the 75-kDa CD9-immunoprecipitated protein

**Peptides**	**Amino-acid location in mortalin**
LYSPSQIGAFVLMK	160–173
MKETAENYLGHTAK	174–187
NAVITVPAYFNDSQR	188–202
VINEPTAAALAYGLDK	219–234
ETGVDLTKDNMALQR	293–307
AQFEGIVTDLIRR	349–361
VQQTVQDLFGR	395–405
LLGQFTLIGIPPAPR	499–513
LFEMAYK	647–653
